# Prognostic significance of collagen signatures in pancreatic ductal adenocarcinoma obtained from second-harmonic generation imaging

**DOI:** 10.1186/s12885-024-12412-5

**Published:** 2024-05-29

**Authors:** Xiwen Chen, Linying Chen, Jikui Miao, Xingxin Huang, Xiahui Han, Liqin Zheng, Shuoyu Xu, Jianxin Chen, Lianhuang Li

**Affiliations:** 1 Key Laboratory of OptoElectronic Science and Technology for Medicine of Ministry of Education, Fujian Provincial Key Laboratory of Photonics Technology, College of Photonic and Electronic Engineering, Fujian Normal University, Fuzhou, 350007 China; 2 Department of Pathology, the First Affiliated Hospital of Fujian Medical University, Fuzhou, 350001 China; 3 Department of General Surgery, Nanfang Hospital, Southern Medical University, Guangzhou, 510515 China

**Keywords:** Second-harmonic generation, Two-photon excited fluorescence, Multiphoton microscopy, Pancreatic ductal adenocarcinoma

## Abstract

**Background:**

Pancreatic ductal adenocarcinoma (PDAC) ranks among the deadliest types of cancer, and it will be meaningful to search for new biomarkers with prognostic value to help clinicians tailor therapeutic strategies.

**Methods:**

Here we tried to use an advanced optical imaging technique, multiphoton microscopy (MPM) combining second-harmonic generation (SHG) and two-photon excited fluorescence (TPEF) imaging, for the label-free detection of PDAC tissues from a cohort of 149 patients. An automated image processing method was used to extract collagen features from SHG images and the Kaplan-Meier survival analysis and Cox proportional hazards regression were used to assess the prognostic value of collagen signatures.

**Results:**

SHG images clearly show the different characteristics of collagen fibers in tumor microenvironment. We gained eight collagen morphological features, and a Feature-score was derived for each patient by the combination of these features using ridge regression. Statistical analyses reveal that Feature-score is an independent factor, and can predict the overall survival of PDAC patients as well as provide well risk stratification.

**Conclusions:**

SHG imaging technique can potentially be a tool for the accurate diagnosis of PDAC, and this optical biomarker (Feature-score) may help clinicians make more approximate treatment decisions.

## Background

Pancreatic cancer has a complex mechanism, high mortality rate, and an extremely poor prognosis [1]. Approximately 90% of pancreatic cancer are pancreatic ductal adenocarcinoma (PDAC) [2]. Its onset is insidious, and most patients are already in the advanced stages when they are diagnosed and still have low 5-year survival rate [3] though genomic screen has been used to obtain functional targets for increased therapeutic effectiveness in pancreatic cancer [4]. Factors that impact survival rates for PDAC include age, gender, underlying health conditions, lifestyle habits, microbiomes, et al., and tumor stage at diagnosis is the most important factor affecting the outcome of PDAC [5, 6]. Identifying new prognostic markers to screen high-risk groups is a novel approach to enhance pancreatic cancer prognosis. Recently, new biomarkers with prognostic significance have been discovered for the management of this disease such as carbohydrate antigen 19 − 9 (CA19-9) [7], haemoglobin A1c [5], laminin-5 gamma-2 (LAMC2) [8], long non-coding RNAs (lncRNAs) [9] and extracellular vesicles (EVs) [10]. One of the most notable features of PDAC is the dense desmoplastic stroma, which is now highly valued and being studied alongside the malignant epithelial component of PDAC. The extracellular matrix (ECM), a prominent element of the PDAC stroma, provides physical and chemical signals that govern the actions of cancerous cells [11, 12]. Irregular ECM in tumor microenvironment would facilitate cancer progression by directly spurring cell transformation and metastasis as well as affecting stromal cell activities such as angiogenesis and inflammation, which can contribute to the composition of tumor microenvironment.

Collagen fibers, which account for approximately 90% or more of the total ECM mass [13], could be used to distinguish normal pancreatic tissues from PDAC tissues. The abundance of collagen fibers throughout the stroma strongly correlates with the progression of PDAC. Interstitial fibrosis in the microenvironment of pancreatic cancer is considered the primary culprit in impeding chemotherapeutic drug delivery. The degradation of collagen fibers contributes to enhanced drug delivery and perfusion [14]. The ECM, which contains ample collagen fibers, not only forms a physical barrier that obstructs the efficacy of chemotherapeutic drugs but also promotes drug resistance in cancer cells through multiple pathways [13]. Some studies suggested that collagen fibers could function as a “highway” and facilitate cancer cell migration and metastasis rather than hindering it [15], whereas some indicated that collagen fibers could act as a barrier to tumor invasion and thus prevent tumor cells from spreading [16]. Currently, hematoxylin and eosin (H&E) staining and Masson’s trichrome staining are the gold standard for the diagnosis of collagen features in PDAC, but these techniques are cumbersome, labor-intensive, and time-consuming. Multiphoton microscopy (MPM) is an optical imaging technique that has the ability to perform label-free imaging with high resolution and low photobleaching [17]. The endogenous signals in biological tissues mainly come from nicotinamide adenine dinucleotide (NADH) and its phosphate derivative (NADPH), flavin adenine dinucleotide (FAD), collagen, elastin, keratin and so on. Therefore, MPM can provide high-contrast images of cells as well as extracellular mesenchyme [18, 19]. This capability helps basic and clinical researchers acquire detailed microstructural information of biological tissues [20], and previous studies demonstrated that MPM imaging could offer significant advantages in cancer detection, disease process research and the qualitative and quantitative analysis of collagen fibers within tumor microenvironment [21–23].

In this work, we made an attempt to introduce MPM combining second-harmonic generation (SHG) and two-photon excited fluorescence (TPEF) imaging to label-free detect PDAC tissues, and found that the characteristics of collagen fibers are diverse. Then, we paid attention to quantitatively analyze the variations in collagen morphological features using an automatic image processing technique, and extracted 8 collagen features from SHG images. We also developed a Feature-score combining the 8 collagen features by ridge regression, and employed it to establish a predictive model of overall survival. Our results demonstrate that SHG imaging can label-free observe the collagen microstructural characteristics of PDAC, and the Feature-score exhibits good prognostic predictive ability for pancreatic cancer patients.

## Materials and methods

### Sample preparation

In this research, all samples were obtained under protocols approved by the Institutional Review Board of the First Affiliated Hospital of Fujian Medical University. We collected 149 Formalin-fixed paraffin-embedded (FFPE) pancreatic cancer tissue samples from 149 patients who were diagnosed between 2010 and 2019. All samples were subjected to standard pathological processing, including formalin fixation, alcohol dehydration, and paraffin embedding. Each paraffin block sample was cut into two 5 μm thick serial tissue sections in the pathology laboratory using an Ultra-Thin Semiautomated Microtome. All sections were deparaffinized with alcohol and xylene before imaging, and one section was stained with hematoxylin and eosin (H&E) and examined histologically under a standard light microscope and the adjacent section was used for MPM imaging.

### Multiphoton microscopic imaging system

The MPM imaging system used in this study is composed of a laser scanning microscope (Zeiss LSM 880, Jena, Germany) and an external mode-locked Ti: Sapphire laser tunable from 690 nm to 1064 nm (Chameleon Ultra, Coherent, USA). We used a laser with a wavelength of 810 nm as the excitation light source, and used a 20× objective lens (Plan-Apochromat, NA = 0.8, Zeiss, Germany) to obtain high-contrast MPM images. In our experiments, two independent channels were used to receive back-facing SHG and TPEF signals at the same time, where the detection wavelength range of SHG signal (green color-coded) was 395–415 nm, and the detection wavelength range of TPEF signal (red color-coded) was 428–695 nm. Additionally, all images are automatically recorded and stitched by Zeiss software after twice averaging, and the pixel depth of the images is 12 bits.

### Collagen morphological features

**Fig. 1:**
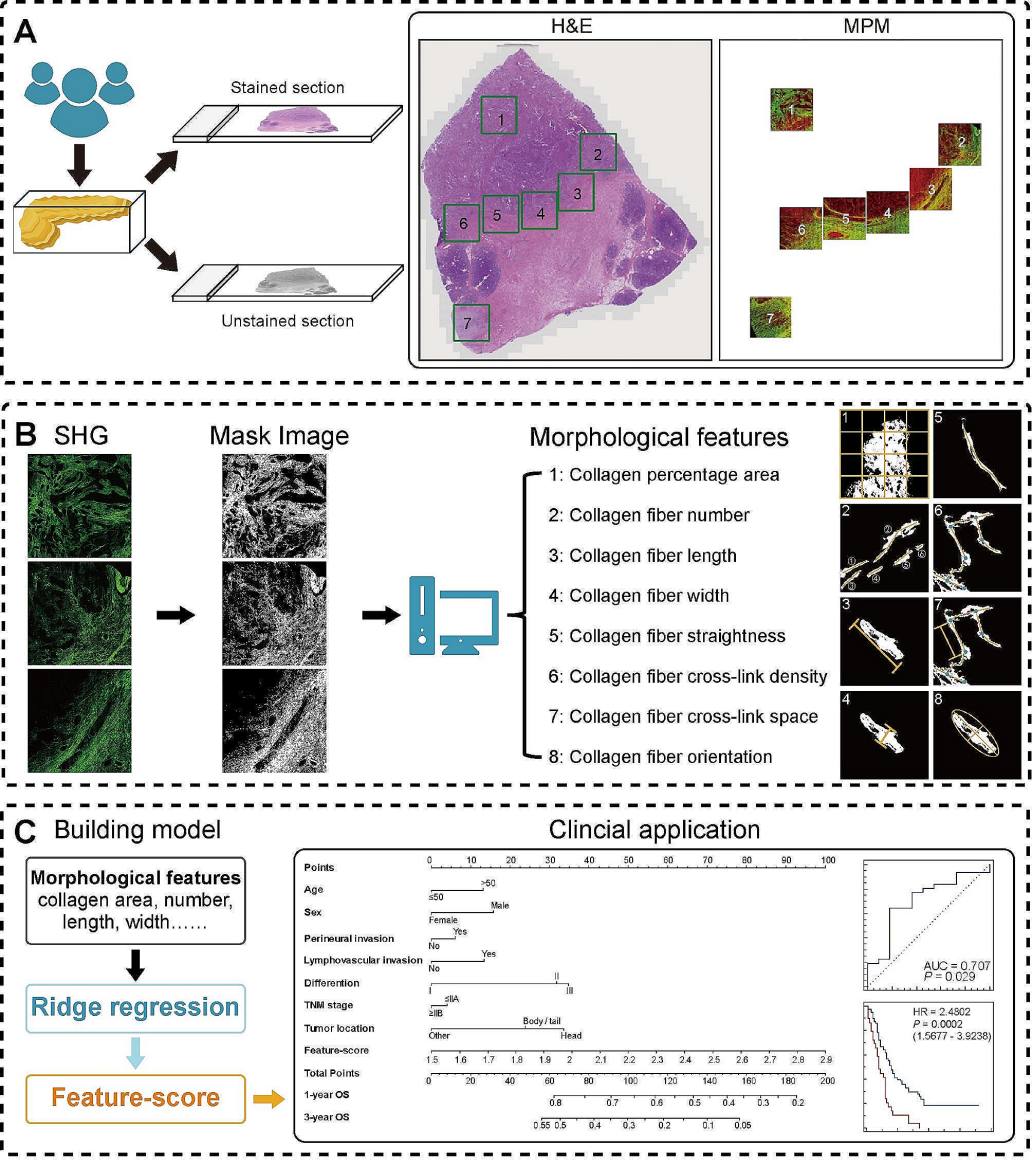
Workflow in this study. Abbreviations: H&E, hematoxylin and eosin; MPM, multiphoton microscopy; SHG, second harmonic generation

In the H&E image of a whole tissue section, we numbered several nonoverlapping regions of interest (ROIs) across the invasive margins and adjacent tumor areas, and performed MPM imaging on all numbered areas on another section (Fig. 1A). Then, we developed an automatic image processing method to quantitatively analyze collagen changes in SHG images [24, 25]: a Gaussian mixture model-based segmentation algorithm was used to segment SHG images into collagen fibers and background; afterwards, to track each fiber, we used a mature fiber network extraction algorithm to process the binary mask image of collagen fibers. After fiber extraction, the skeleton of each fiber was identified and represented by a list of ordered vertices, and if any vertex in the list belongs to more than one fiber, it would be identified as a cross-link point. The list of vertices was used to calculate fiber number, length, width, straightness, cross-link density, and cross-link spacing. In addition, the orientation of collagen alignment was quantified using Fourier transform. A lot of 8 collagen morphological features as previously described including collagen proportionate area (fea1), fiber number (fea2), fiber length (fea3), fiber width (fea4), fiber straightness (fea5), fiber cross-link density (fea6), collagen fiber cross-link spacing (fea7), and collagen fiber orientation (fea8), were extracted from SHG images using MATLAB 2016b, and all ROIs were averaged to generate 8 morphological values for each patient (Fig. 1B). In this work, we choose a total of 75,647 SHG images with 512*512 pixels for quantitative analysis. Finally, we used ridge regression with cross validation to retrieve the coefficient of fea1-8 in the training cohort (Table 1), and then a Feature-score was obtained for each patient by a linear combination of the 8 features weighted by their regression coefficients, where.

Feature-score=-7.7656606*fea1-44.6439289*fea2 + 0.309178*fea3 + 0.8884292*fea4-1.1665562*fea5 + 10.1583025*fea6-0.1356464*fea7 + 0.6020531*fea8.

### Statistical analysis

All statistical analysis was performed with R 4.2.2 and IBM SPSS Statistics 24. We conducted all statistical tests by a two-sided basis, and *P*-value less than 0.05 was considered statistically significant. For survival analysis, overall survival (OS) was used as the endpoint, defined as the time from diagnosis to death, or if the patient was enrolled, from diagnosis to the end of follow-up. The independent predictors were selected using univariate and multivariate Cox proportional hazard regressions. A nomogram was constructed using all risk factors to estimate OS, and the calibration of nomogram was evaluated by a calibration plot, which was a graphic representation of the relationship between the actual incidence and predicted probability. We also performed receiver operating characteristic (ROC) curve analysis to calculate the areas under the curves (AUCs) for estimating predictive accuracy, and to determine the optimal cutoff value by maximizing the Youden index in the training cohort which was also applied to the validation cohort. Moreover, the Kaplan-Meier survival curve was used to assess the correlation between variables and OS.

## Results

### Baseline clinical characteristics

For this study, 149 patients were divided into training (107 cases) and validation (42 cases) cohorts. Table 1 shows the clinical characteristics of patients in the two cohorts. Baseline clinicopathologic data of each patient, including age of diagnosis, sex, TNM stage, differentiation, perineural invasion, lymphvascular invasion and tumor location were collected. The average age of patients at the diagnosis of pancreatic cancer was 60.9 years (median, 61 years; range, 28–86 years), and the median follow-up duration was 13 months (range, 1–72 months) for OS.

**Table 1:** Characteristics of patients with pancreatic ductal adenocarcinoma (PDAC)

Characteristics	Training cohort (107)	Validation cohort (42)	*p*	Total cohort (149)
**Age (%)**			0.262	
<=50	17 (15.9)	12 (28.6)		29 (19.5)
> 50	90 (84.1)	30 (71.4)		120 (80.5)
**Sex (%)**			0.982	
Male	69 (64.5)	25 (59.5)		94 (63.1)
Female	38 (35.5)	17 (40.5)		55 (36.9)
**TNM stage (%)**			0.364	
<=IIA	47 (43.9)	15 (35.7)		62 (41.6)
>=IIB	60 (56.1)	27 (64.3)		87 (58.4)
**Differentiation (%)**			0.316	
I	16 (15.0)	12 (28.6)		28 (18.8)
II	45 (42.0)	17 (40.5)		62 (41.6)
III	46 (43.0)	13 (30.9)		59 (39.6)
**Perineural invasion (%)**			0.761	
Yes	84 (78.5)	30 (71.4)		114 (76.5)
No	23 (21.5)	12 (28.6)		35 (23.5)
**Lymphovascular invasion (%)**			0.527	
Yes	20 (18.7)	4 (9.5)		24 (16.1)
No	87 (81.3)	38 (90.5)		125 (83.9)
**Tumor location (%)**			0.672	
Head	75 (70.1)	32 (76.2)		107 (71.8)
Body/tail	29 (27.1)	8 (19.0)		37 (24.8)
Other	3 (2.8)	2 (4.8)		5 (3.4)

### Identifying collagen morphological features of PDAC via MPM

**Fig. 2:**
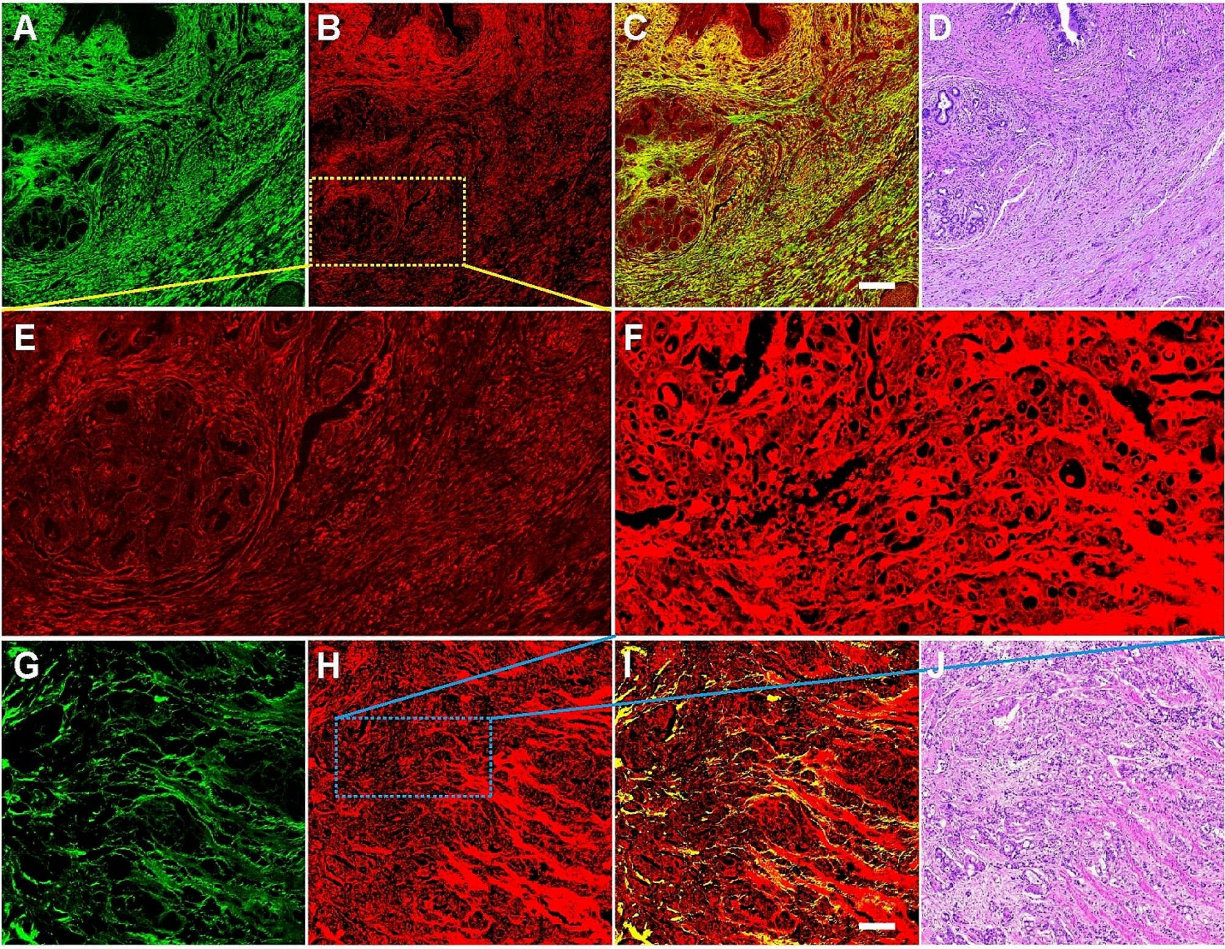
MPM images and the corresponding H&E-stained images of PDAC. (A, G) SHG images; (B, H) TPEF images; (C, I) Overlap images of SHG and TPEF; (D, J) H&E-stained images; (E, F) Magnified images of the regions of interest (yellow and blue dashed boxes, respectively). Scale bar = 200 μm

The accurate diagnosis of PDAC is currently based on histopathological examination, however, this is a labor-intensive and time-consuming process. Several previous studies have used MPM to image and analyze normal and PDAC tissues [26, 27]. In this work, SHG and TPEF imaging were combined to image PDAC tissues to detect histological changes in tumor microenvironment. As shown in Fig. 2, the architectural features of PDAC are clearly present in the MPM images, where SHG imaging (Fig. 2A, G) shows the distribution of collagen fibers, while TPEF imaging (Fig. 2B, H) is able to identify tumors, and composite images (Fig. 2C, I) allow the user to visualize the spatial distribution of tissue components, and magnified images (Fig. 2E, F) of the regions of interest (yellow and blue dashed boxes, respectively) show tumors cells more clearly. All these features correspond to the H&E‑stained images (Fig. 2D, J). Interestingly, SHG images reveal the different characteristics of collagen fibers in tumor microenvironment: some collagen fibers are an ordered, abundant and have directed distribution (Fig. 2A), and by contrast, some are sparse, disordered, and fragmented (Fig. 2G).

**Fig. 3:**
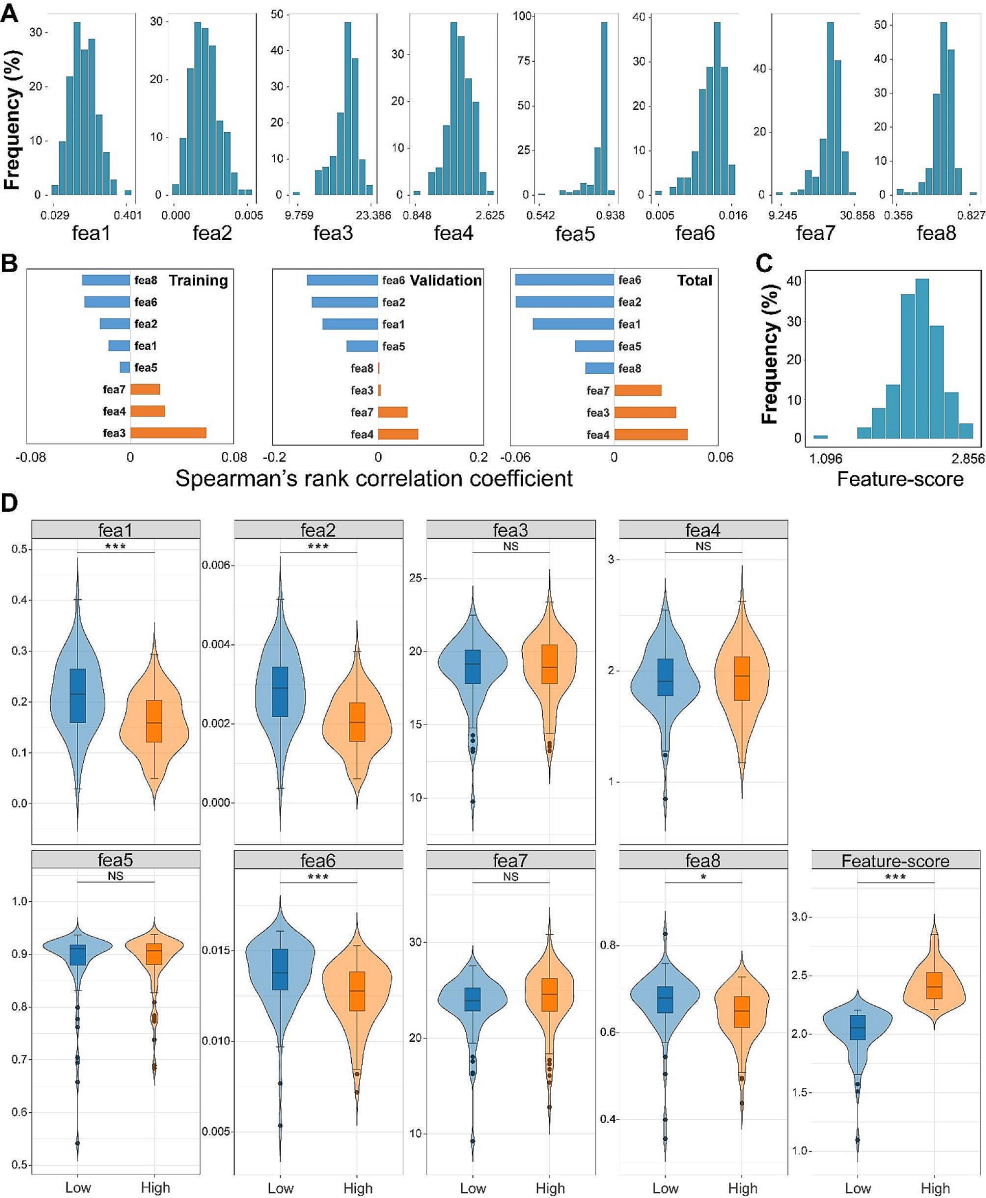
(**A**) Distribution histograms of collagen features; (**B**) Correlation analysis between individual collagen feature and OS for three cohorts; (**C**) Distribution histogram of Feature-score; (**D**) Violin plots of collagen features and Feature-score to display data distribution between high-risk and low-risk groups. Fea1: collagen proportionate area; fea2: fiber number; fea3: fiber length; fea4: fiber width; fea5: fiber straightness; fea6: fiber cross-link density; fea7: collagen fiber cross-link spacing; fea8: collagen fiber orientation; *P < 0.05; **P < 0.01; ***P < 0.001; NS: Not significant

Previous studies have shown a link between cancer progression and collagen changes in the tumor microenvironment [23, 28]. Therefore, to quantify these collagen morphological differences, we performed image analysis of SHG images and obtained 8 values of collagen features (fea1-8). The distribution histogram of fea1-8, including fiber area, number, length, width, orientation, straightness, cross‑link spacing, and cross‑link density, are presented in Fig. 3A. We found that fea1, 2, 4, 6 were evenly distributed, whereas fea3, 5, 7, 8 were unevenly distributed. The correlation between individual collagen feature and OS was also assessed by Spearman’s rank correlation coefficient. The results reveal that fea1, 2, 5, 6 correlate with poor overall survival, while fea3, 4, 7 correlate with good overall survival (Fig. 3B). To reduce the disadvantages caused by uneven distribution, a ridge regression was performed based on the 8 feature values from training set (54,735 SHG images) to obtain a formula of Feature-score, which was then used to calculate the Feature-sore of each patient. As shown in Fig. 3C, the distribution of Feature-score is more uniform compared to individual collagen features. Thus, it is reasonable to comprehensively consider the effect of all collagen features.

### Prognosis predictive performance of Feature-score

**Fig. 4:**
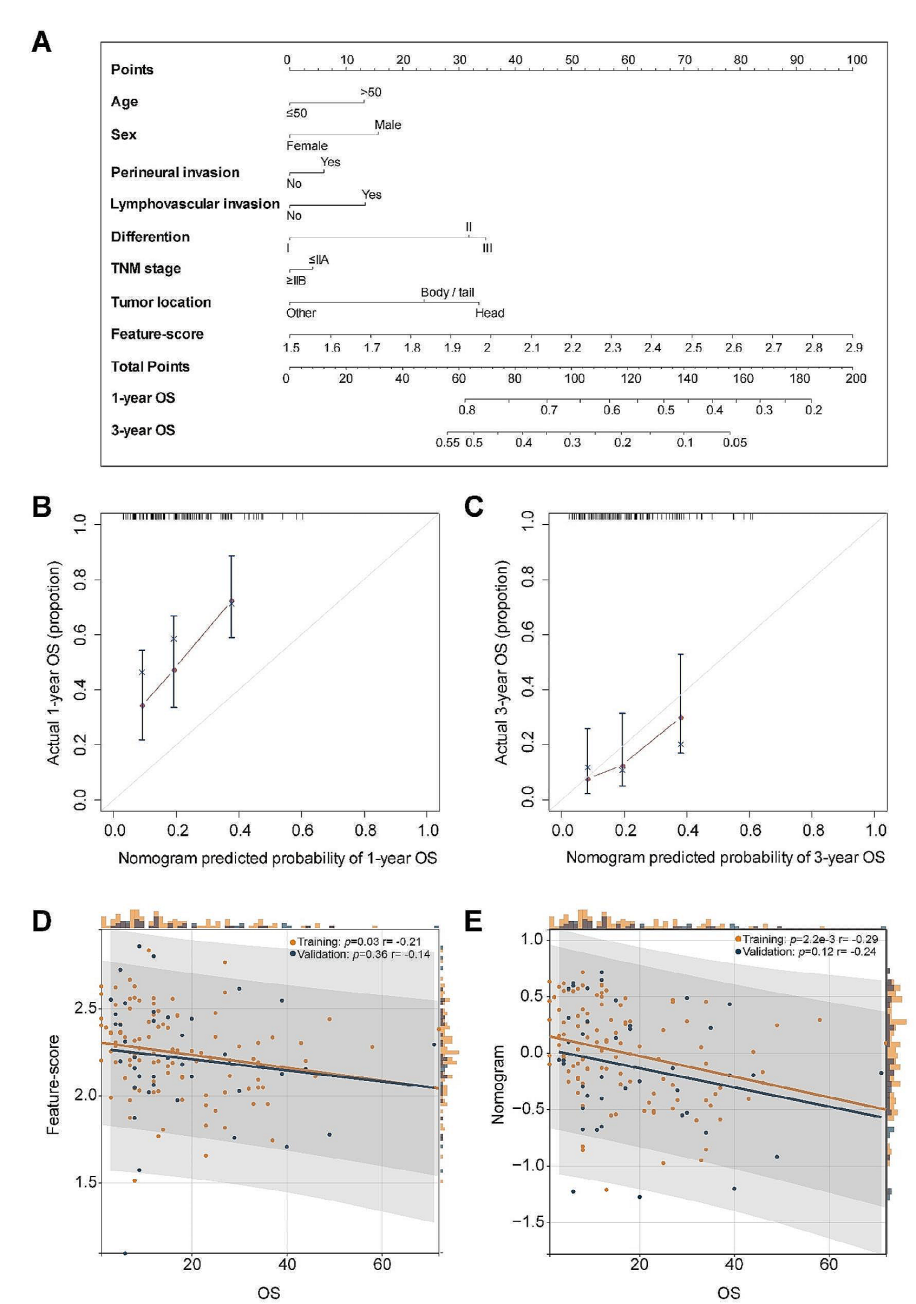
(**A**) Nomogram combining Feature-score, age, sex, perineural invasion, lymphovascular invasion, TNM stage, differentiation and tumor location from the training cohort; (**B**-**C**) Calibration curves of the nomogram to predict 1-year and 3-year OS rate respectively from training cohort; (**D**-**E**) Scatter plots presenting the correlation of Feature-score and nomogram model with OS, and histogram plots above the scatter plots show OS, while the plots in the right of the scatter plots show the Feature-score and nomogram model and the correction coefficient (*r*) is obtained by performing Pearson’s correction analysis

As shown in Table 2, the Feature-score is significantly associate with OS in univariate Cox proportional hazard regression analysis (HR 2.984, [1.429–6.230], *P* = 0.004). After adjusting for clinical variables by multivariate Cox regression analysis, the Feature-score remains an independent prognostic indicator for predicting OS (HR 2.562, [1.190–5.513], *P* = 0.016) in the whole cohort. Noteworthy, collagen morphological features remain the most significant factors (with the smallest *P* values) compared to the clinical factors. Moreover, a nomogram model containing Feature-score, age, sex, TNM stage, differentiation, perineural invasion, lymphvascular invasion and tumor location was established from training cohort (Fig. 4A), wherein each prognosticator is given a point based on the corresponding point scale, and the sum of total points of all prognosticators is used to predict the survival of PDAC patients. We can see that the Feature-score considerably outweighs clinical factors. As shown in Fig. 4B and C, the calibration curves reveal improved consistency between the nomogram-estimated probability and observed probability for 3-year OS as opposed to 1-year OS. Correlation analysis demonstrates that low Feature-score corresponds to long OS (Fig. 4D) and similar result was observed in nomogram model, but nomogram shows better correlation and significance compared with the Feature-score model (training cohort, correlation coefficient [*r*], 0.29 vs. 0.21; validation cohort, [*r*], 0.24 vs. 0.14) (Fig. 4D and E).

**Table 2:** Univariate and multivariate Cox proportional hazards regression analysis to analyze the relationship between prognostic factors and OS in patients with PDAC

Characteristics	Univariate analysis					Multivariate analysis			
	HR	(95%CI)		*P*-value		HR	(95%CI)		*P*-value
**Age**									
≤ 50	Reference								
> 50	0.788	0.501	1.238	0.301		NA			NA
**Sex**									
Female	Reference								
Male	1.204	0.821	1.764	0.342		NA			NA
**Perineural invasion**									
No	Reference								
Yes	1.106	0.719	1.700	0.647		NA			NA
**Lymphovascular invasion**									
No	Reference								
Yes	1.765	1.124	2.771	0.014		1.471	0.924	2.341	0.104
**TNM stage**									
<=IIA	Reference								
>=IIB	1.148	0.796	1.656	0.460		NA			NA
**Differentiation**									
I	Reference								
II	1.763	1.033	3.009	0.038		1.471	0.849	2.547	0.168
III	1.759	1.039	2.980	0.036		1.541	0.985	2.652	0.119
**Tumor location**									
Head	Reference								
Body/tail	1.408	0.929	2.132	0.230		NA			NA
Other	1.058	0.387	2.894	0.912		NA			NA
**Feature-score**	2.984	1.429	6.230	0.004		2.562	1.190	5.513	0.016

NA: Not applicable

**Fig. 5:**
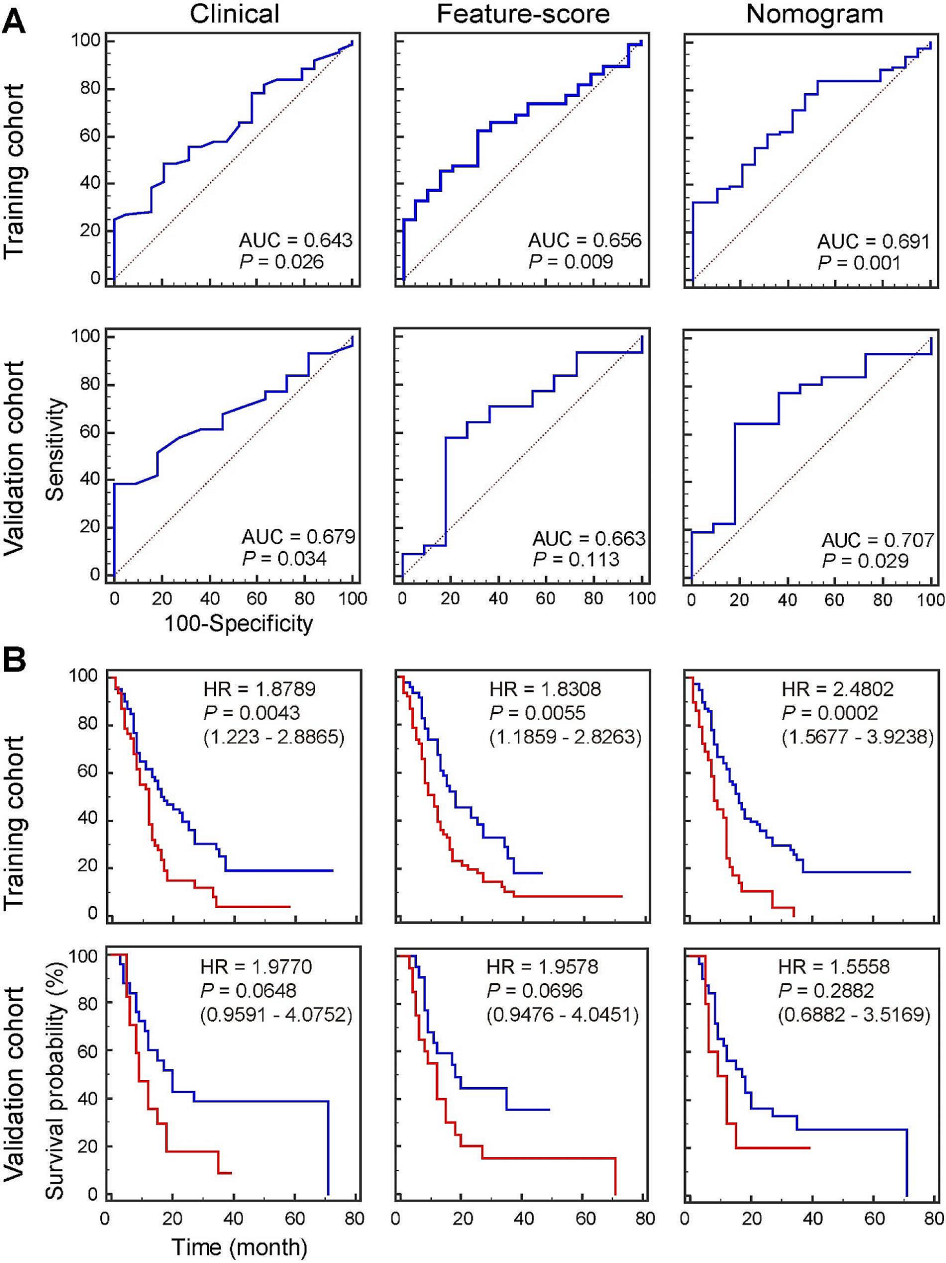
(**A**) ROC curves of the clinical, Feature-score and nomogram models to predict OS in the training and validation cohorts; (**B**) Kaplan-Meier curves of OS according to the clinical, Feature-score and nomogram models in the training and validation cohorts. The red lines indicate high risk and blue lines indicate low risk in the Kaplan-Meier curves. A two-sided log-rank test was performed to determine statistical significance. Abbreviations: OS, overall survival; HR, hazard ratio

The AUC of Feature-score was further calculated to assess its predictive accuracy, and a clinical (CLI) model combining the seven clinical factors was also developed for comparison. As depicted in Fig. 5A, the CLI model reveals an AUC of 0.643 (95% CI, 0.517 to 0.768) in the training cohort and Feature-score gains an AUC of 0.656 (95% CI, 0.538 to 0.773) for predicting OS, demonstrating that the Feature-score as an optical biomarker has equal predictive capability compared to the CLI model, and nomogram model combining the Feature-score and CLI model could improve the AUC to 0.691 (95% CI, 0.574 to 0.809). As anticipated, similar findings are observed in the validation cohort, thereby confirming the dependability of this score. We could also use the optimal cutoff values (Youden index: CLI model is 0.5834; Feature-score model is 2.2043; Nomogram model is 0.2917) of three predictive models for identifying high-risk and low-risk patients in both the training and validation cohorts, and then use the Kaplan-Meier survival curve to perform survival analysis. As shown in Fig. 5B, the CLI, Feature-score and nomogram models deliver an increasing HR from 1.8 to 2.5 in training cohort, indicating an enhanced ability for risk stratification, but similar results are not obtained in validation cohort. In addition, violin plots of the eight collagen features (fea1-8) and Feature-score of the whole cohort were presented in Fig. 3D to display the data distribution between high-risk and low-risk groups, and statistical analyses show obvious difference (*P* < 0.05) in collagen proportionate area, collagen fiber number, cross-link density, orientation and Feature-score.

## Discussion

PDAC is the fourth leading cause of cancer deaths, and it is estimated that it will become the second leading cause of cancer deaths after lung cancer by 2030 [29]. PDAC is a grave malignant tumor with a mortality rate that is tightly correlated with its incidence. It is typically asymptomatic until the cancer has progressed to an advanced stage [30]. Despite the progress made in detecting PDAC, the prognosis remains unsatisfactory, with a five-year survival rate of just 12%. Most patients were diagnosed when malignant, and only a few can expect to receive surgery [31, 32]. At present, histological examination of the resected specimens is still the gold standard for diagnosing PDAC, but it requires staining and much time and effort. Therefore, the implementation of a better auxiliary diagnostic approach would have significant impact. MPM is a powerful tool in biomedical imaging, capable of capturing cells and extracellular matrix in tissues, providing exceptional resolution and imaging capability. Bodelon et al. utilized this optical technique to investigate collagen fiber profiles in breast and discovered that collagen profiles were linked with the risk of breast cancer [33]; Rogart et al. proved that MPM has the capacity to scrutinize gastrointestinal mucosa at the cellular level [34]; Cromey et al. used multiphoton imaging technique to rapidly differentiate normal tissues from pancreatic cancer [35]; and Gomes et al. combined multiphoton imaging with an automated image analysis to successfully recognise and quantify stromal fibers and neoplastic cell regions from MPM images of prostate cancer tissues [36]. Our study also indicates that MPM can label-free identify PDAC and thereby can assist in prognostic study.

One of the most notable characteristics of PDAC is its considerable dense connective proliferative stroma. Many previous studies have proved a close relationship between collagen distribution and various diseases: Drifka et al. revealed that a high alignment of stromal collagen fibers was linked to an unfavorable prognosis in patients with PDAC [37]; Li et al. demonstrated that different subtypes of ovarian cancer had distinctive collagen properties by analyzing SHG images [21]; and Garcia et al. found that there was a clear association of collagen fiber length with the survival times of dogs by image analyses, the canine mammary carcinomas presenting shorter collagen fibers indicate a worse survival rate [38]. We have combined two-photon microscopy with imaging analysis to study gastrointestinal stromal tumors (GISTs) and found that there are collagen morphological differences (fiber area, density and cross-link density) between normal and tumor tissues [24]. Moreover, we have also used a computer-assisted image processing method to automatically extract tumor-associated collagen microscopic signatures from SHG images of breast cancer and then provided a collagen-related score (TCMF-score) for each patient, and statistical result demonstrated that TCMF-score is an independent prognostic factor [25]. Thus, in this study, we focused on detecting collagen changes in tumor microenvironment by SHG imaging and aimed to investigate the prognostic significance of collagen features in PDAC. Imaging results show that SHG images can clearly illustrate the morphology and spatial distribution of collagen fibers in PDAC. We easily found the different variations in collagen morphology, and to quantify these results, we further extracted eight collagen features from the SHG images and used ridge regression to acquire a Feature-score based on these feature values. Statistical analysis reveals that Feature-score, as an independent factor, enables us to equally predict the overall survival of patients with PDAC in contrast to the CLI model combining 7 clinical factors (close AUC values), and has comparable risk stratification. Our finding also indicates that higher Feature-score corresponds to decreased survival rate in PDAC patients. A nomogram model, which could improve the discrimination (higher AUC), was also established to individually evaluate the OS rate of PDAC patients. This scoring model demonstrates the potential value of collagen signatures in PDAC, and may be useful in determining individual treatment strategies. Of course, we recognize that this study has some limitations: firstly, the sample size is small; secondly, it is a retrospective study in a single center. We need to collect more samples to refine the Feature-score model and carry out multiple-center validation in the next work.

## Conclusions

In short, we tried to use multiphoton imaging to label-free detect PDAC tissues, and our findings demonstrate the effectiveness of SHG imaging as a powerful tool for capturing collagen changes in tumor microenvironment. Then, we extracted eight microstructural features of collagen fibers from SHG images and obtained a Feature-score by combining these features using ridge regression. Statistical results reveal that as an independent factor, a Feature-score could effectively predict the overall survival of PDAC patients and also has well performance in risk stratification. The combination of SHG imaging technique and quantitative information may be helpful in diagnosing PDAC and making reasonable treatment strategy.

## Data Availability

Data are available from the corresponding authors on reasonable request.

## Abbreviations

SHG: Second Harmonic Generation
TPEF: Two-Photon Excited Fluorescence
MPM: Multiphoton Microscopy
PDAC: Pancreatic Ductal Adenocarcinoma
ECM: Extracellular Matrix
FAD: Flavin Adenine Dinucleotide
NADH: Nicotinamide Adenine Dinucleotide
NADPH: Nicotinamide Adenine Dinucleotide Phosphate
H&E: Hematoxylin and Eosin
ROI: Region Of Interest
OS: Overall Survival
AUC: Area Under the Curve
HR: Hazard Ratio
